# Mosquitos Again

**Published:** 1900-06-30

**Authors:** 


### MOSQUITOS AGAIN.

In a report on the occurrence of malaria and other sub tropical diseases in the Dominion of Canada, addressed to the Secretary of State for the Colonies by Professor Adami, of Montreal, the interesting observation is recorded that, just as in Great Britain, even to the middle of this century, cases of malaria occurred with considerable frequency, so in Canada, within the memory of inhabitants still living, malaria was not infrequent along the St. Lawrence, even in the neighbourhood of Montreal. Now, however, it is only in the most southerly portion of Canada, namely, in the province of Ontario, between the Great Lakes, that malaria is met with, and even there not to any great extent. Nevertheless, mosquitos abound in extraordinary numbers, and members of the genus Anopheles are still present; and Professor Adami urges with great force that in view of the extraordinary abundance of lakes and large bodies of water, and the relatively small proportion of the country that has undergone full drainage and cultivation, the gradual disappearance of the disease is a problem difficult to solve. He states that during the seven years he has been pathologist to the two largest hospitals in Montreal he has only come across one case of malaria in one who had not been out of the country. Of course, one recognises that mosquitos may exist in any quantity without malaria, so long as the malarial infection is not present in man, but malaria when already present, as was here the case, ought not, according to modern theories, to die out in the presence of infective material when the necessary mosquitos exist in such enormous multitudes; and Professor Adami argues, with apparent reason, that the fact of the practical cessation of malaria, while the mosquitos continue in full force, shows that some other conditions besides the presence of the hosts of the malarial micro-organism would seem necessary for the persistence and propagation of the malarial germ. The observation is important since it seems to go directly against much recent teaching.

